# Durlobactam, a Broad-Spectrum Serine β-lactamase Inhibitor, Restores Sulbactam Activity Against *Acinetobacter* Species

**DOI:** 10.1093/cid/ciad095

**Published:** 2023-05-01

**Authors:** Krisztina M Papp-Wallace, Sarah M McLeod, Alita A Miller

**Affiliations:** Research Service, Veterans Affairs Northeast Ohio Healthcare System, USA; Departments of Biochemistry and Medicine, Case Western Reserve University, Cleveland, OH, USA; Entasis Therapeutics, Waltham, MA, USA; Entasis Therapeutics, Waltham, MA, USA

**Keywords:** *Acinetobacter*, sulbactam, durlobactam, β-lactamase inhibitor, diazabicyclooctane

## Abstract

Sulbactam-durlobactam is a pathogen-targeted β-lactam/β-lactamase inhibitor combination in late-stage development for the treatment of *Acinetobacter* infections, including those caused by multidrug-resistant strains. Durlobactam is a member of the diazabicyclooctane class of β-lactamase inhibitors with broad-spectrum serine β-lactamase activity. Sulbactam is a first-generation, narrow-spectrum β-lactamase inhibitor that also has intrinsic antibacterial activity against *Acinetobacter* spp. due to its ability to inhibit penicillin-binding proteins 1 and 3. The clinical utility of sulbactam for the treatment of contemporary *Acinetobacter* infections has been eroded over the last decades due to its susceptibility to cleavage by numerous β-lactamases present in this species. However, when combined with durlobactam, the activity of sulbactam is restored against this problematic pathogen. The following summary describes what is known about the molecular drivers of activity and resistance as well as results from surveillance and in vivo efficacy studies for this novel combination.

Sulbactam was designed and synthesized by Pfizer in the late 1970s with the objective of inhibiting β-lactamases when given in combination with ampicillin [1]. This penicillanic acid sulfone–based β-lactamase inhibitor, with a limited inhibition spectrum toward certain class A β-lactamases (eg, SHV and CTX-M), was found to lower minimum inhibitory concentrations (MICs) of ampicillin when tested in combination against gram-positive and gram-negative pathogens [1]. By the early 1980s, the intrinsic antimicrobial activity of sulbactam against *Acinetobacter* species was first observed and confirmed later against contemporary clinical isolates of *Acinetobacter* species [2,3]. Although sulbactam alone was not available for clinical use, the ampicillin-sulbactam combination was (and is still) used in the treatment of infections due to *Acinetobacter* species [4–7]. In initial studies using membrane preparations from *Acinetobacter* species, sulbactam was found to inhibit penicillin-binding protein 2 (PBP2), which was suggested to be its primary mechanism of intrinsic activity toward *Acinetobacter* species [8,9]. However, a subsequent study conducted with purified PBPs from *Acinetobacter* species revealed that the half maximal inhibitory concentration for sulbactam was 55 µM and 4 µM for PBP1a and PBP3, respectively; thus, these PBPs are likely targets for sulbactam [10]. Moreover, the inhibition of PBP3, and PBP1 to a lesser extent, by sulbactam was further confirmed in another study by the determination of in vitro acylation rates as well as microscopy that revealed the classical filamentation morphology observed due to PBP3 inhibition of *Acinetobacter* species when treated with sulbactam [11]. Thus, PBP3 is likely the main target for sulbactam in *Acinetobacter* species.

However, resistance to β-lactam-sulbactam combinations in *Acinetobacter* species, especially multidrug- and carbapenem-resistant strains, has increased [12–15]; in one study from Detroit, Michigan, resistance increased from 10% to 60% from 2003 to 2008 [16]. Resistance has been linked to increased expression of TEM-1, ADC-30, and metallo-β-lactamases in *Acinetobacter* species [11,17–19]. A more in-depth analysis of spontaneous resistance in *Acinetobacter* species was conducted by Penwell et al, who found that frequency of resistance to sulbactam is low (1.39 × 10^−9^ to 4.15 × 10^−10^) and is typically associated with mutations in the gene that encodes PBP3 [11]. Additional sulbactam resistance mechanisms identified included those involved in cell wall metabolism or stress responses [11]. Resistance to sulbactam appears to confer a fitness cost as revealed by in vitro growth rates [11]. The ability of sulbactam to be hydrolyzed by different β-lactamases was also assessed and class A, C, and D serine β-lactamases (eg, TEM-1, KPC-2, ADC-7, OXA-23, OXA-24) as well as class B metallo-β-lactamases (eg, New Delhi metallo-β-lactamase [NDM]–1) were able to turn over sulbactam with varying degrees of activity; of the enzymes tested, only SHV-5 was potently inactivated by sulbactam (ie, *k*_inact_/*K*_I_ >100 000 M^−1^s^−1^) [19]. Increased resistance to approved β-lactam–sulbactam combinations as well as other anti-*Acinetobacter* agents has led to increased mortality and the need for novel treatment strategies [20,21]. Because the main mechanism for resistance to sulbactam is the production of β-lactamases, combining sulbactam with a potent β-lactamase inhibitor that covers common class A, C, and D β-lactamases produced by *Acinetobacter* species seemed necessary to preserve its clinical effectiveness.

## DURLOBACTAM IS A DIAZABICYCLOOCTANE β-LACTAMASE INHIBITOR WITH BROAD-SPECTRUM INHIBITION OF SERINE β-LACTAMASES AND PBPs

**Figure 1.**
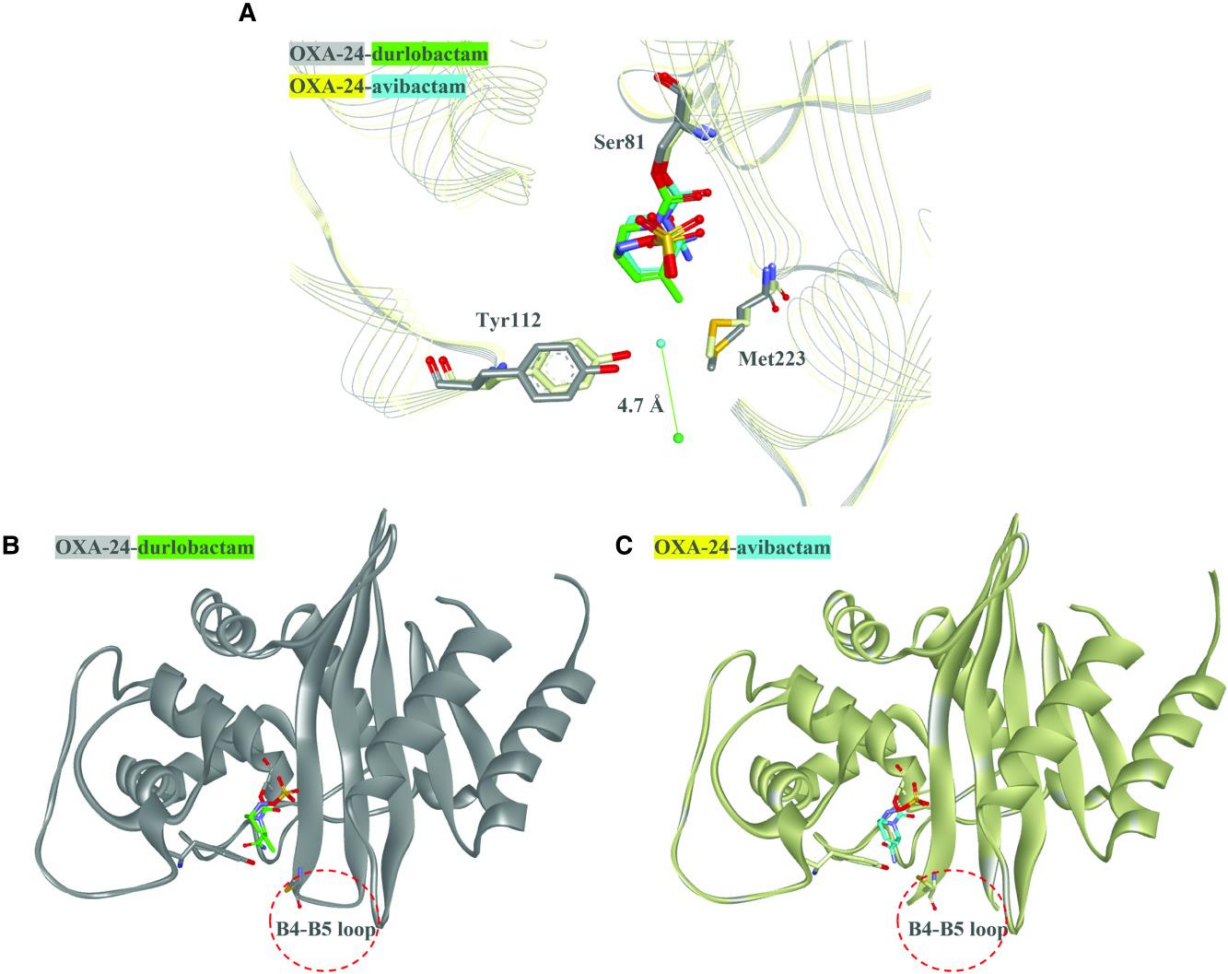
Co-crystal structures of durlobactam or avibactam and OXA-24. *A*, The crystal structures of OXA-24 (gray) with durlobactam (green) (Protein Data Bank [PDB]: 6MPQ) and OXA-24 (yellow) with avibactam (cyan) (PDB: 4WM9) show that the water molecule in the OXA-24–durlobactam structure (green ball) is 4.7Å away from the hydrophobic bridge formed by Tyr112 and Met223 compared to the OXA-24–avibactam structure (cyanball). This difference is likely due to the methyl side chain present on durlobactam that is lacking on avibactam. *B*, The crystal structure of OXA-24 (gray) with durlobactam (green) (PDB: 6MPQ) reveals that the B4-B5 loop is resolved in the structure. *C*, The crystal structure of OXA-24 (yellow) with avibactam (cyan) (PDB: 4WM9) lacks resolution of the B4–B5 loop, suggesting that this region is flexible.

Commercially available β-lactamase inhibitors (eg, avibactam, relebactam, vaborbactam) partnered with different β-lactams demonstrate activity versus class A and C β-lactamases in vitro [22]. Moreover, they are effective antimicrobials against Enterobacterales and *Pseudomonas aeruginosa* with class A and C β-lactamases, but not against most/all class D β-lactamases or *Acinetobacter* species. Thus, durlobactam (formerly ETX2514), a novel diazabicyclooctane (DBO) β-lactamase inhibitor, was rationally designed and synthesized by Entasis Therapeutics to maintain potent class A and C inactivation; however, in addition, using structure–activity relationships, durlobactam's inhibition profile was expanded to include class D β-lactamases (eg, OXA-23, OXA-24, and OXA-58) often produced by *Acinetobacter* species (Table 1) [23,24]. The expanded profile of durlobactam was achieved by modifying the compound for size and polarity, which also enhanced penetration into the bacterial cell and was subsequently found to transverse OmpA in *Acinetobacter* species [23,26]. Also, the addition of the double bond enhanced the reactivity of durlobactam [23]. Crystallography and molecular modeling of class D β-lactamases with durlobactam and other durlobactam-like DBOs, as well as another similar β-lactamase inhibitor, avibactam, which lacks the double bond and methyl side chain, were performed. These studies revealed that durlobactam and durlobactam-like compounds did not disrupt the hydrophobic bridge (Met 223:Tyr112) and blocked solvent from accessing the covalent bond with the nucleophilic serine present in class D β-lactamases, thus likely leading to the improved potency and decreased deacylation, respectively (Figure 1*A* ) [23]. By evaluating the structures published in 2 other studies, the loop between B4 and B5 β-strand of OXA-24 is not resolved in the OXA-24–avibactam structure (Protein Data Bank [PDB]: 4WM9) compared to the OXA-24–durlobactam structure (PDB: 6MPQ), suggesting that the loop is flexible in the former structure; this flexibility may slow the acylation of avibactam compared to durlobactam (Figure 1*B* and 1*C* ) [24,27]. A quantum mechanics/molecular modeling study comparing avibactam and durlobactam versus OXA-24 further revealed that protonation states of Lys84 and Lys218 are crucial for the rapid chemical reactivity of durlobactam over that of avibactam [28]. Durlobactam rapidly inactivates class A, C, and D β-lactamases and is slow to recyclize (ie, reform active inhibitor), with *k*_off_ rates (ie, how quickly durlobactam dissociates from the enzyme) being the lowest for class D enzymes (Table 1) [24,25]. Minor hydrolysis of durlobactam was observed with KPC-2 after a 2-hour incubation; however, these conditions were not considered physiologically relevant [25]. Moreover, durlobactam was also found to be an inhibitor of PBPs, particularly PBP2 (Table 1) [23]. Thus, sulbactam-durlobactam not only inactivates PBP3 and PBP2, respectively, but also inhibits class A, C, and D β-lactamases found in *Acinetobacter* species. The multiple targets of sulbactam-durlobactam likely contribute to its antimicrobial potency as described below.

**Table 1.** Inhibitory Kinetic Parameters for Durlobactam Against Representative β-lactamases and Penicillin-Binding Proteins

β-lactamase/PBP	*k*_2_/*K* (M^−1^s^−1^)	*k*_off_ (s^−1^)
TEM-1 (class A)	1.4 ± 0.6 × 10^7^	1.4 ± 0.2 × 10^−3^
KPC-2 (class A)	9.3 ± 0.6 × 10^5^	1.0 ± 0.1 × 10^−3^
ADC-7 (class C)	1.0 ± 0.1 × 10^6^	8.0 ± 0.1 × 10^−4^
OXA-24 (class D)	9.0 ± 0.2 × 10^3^	1.7 ± 0.1 × 10^−5^
*Acinetobacter baumannii* PBP1a	18 ± 0.6 × 10^1^	Not determined
*A. baumannii* PBP2	1.8 ± 0.6 × 10^3^	Not determined
*A. baumannii* PBP3	3.37 ± 0.06	Not determined

Source: [23–25]. The *k*_2_/*K* value represents the acylation rate, or how quickly durlobactam gets bound to a β-lactamase or PBP; the higher the number, the faster the reaction. The *k*_off_ value indicates how quickly durlobactam dissociates from the β-lactamase; the lower the number, the slower durlobactam comes off the β-lactamase.

Abbreviation: PBP, penicillin-binding protein.

## FREQUENCY AND MECHANISMS OF RESISTANCE TO SULBACTAM-DURLOBACTAM

**Figure 2.**
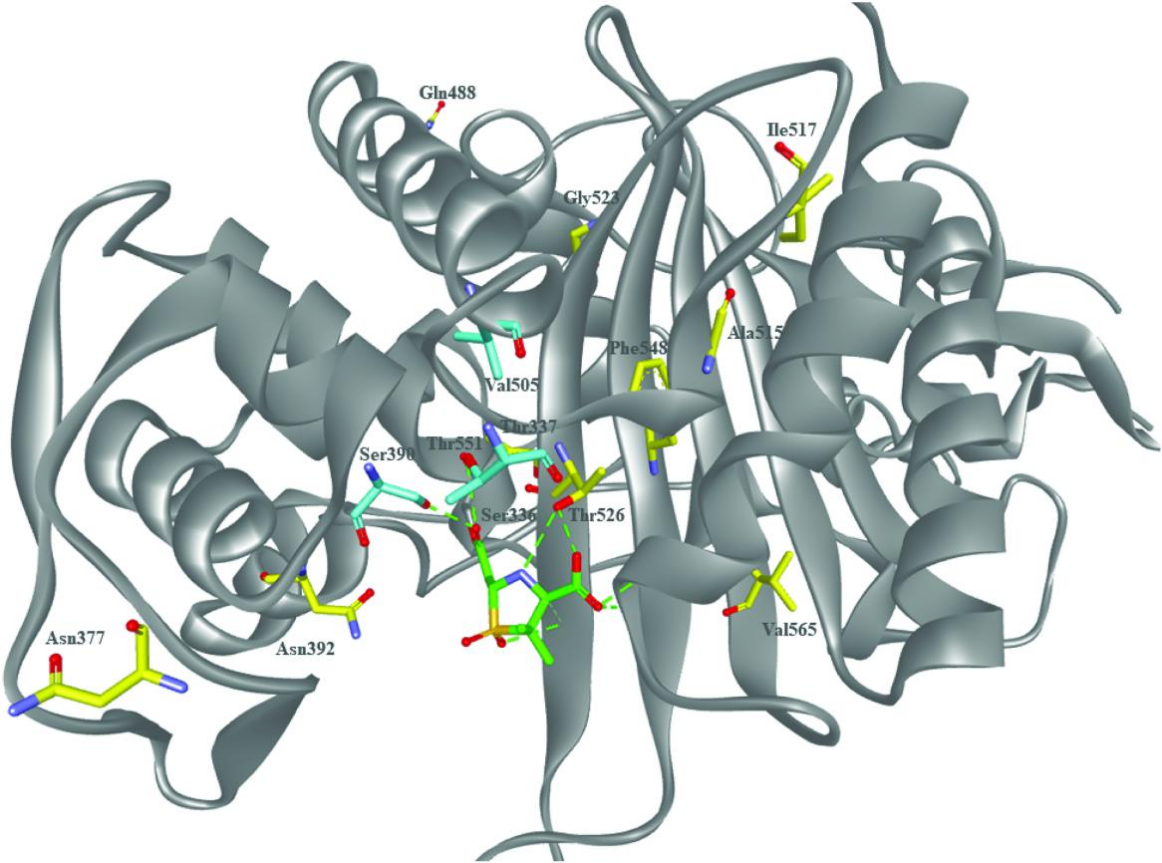
Molecular model of sulbactam bound to *Acinetobacter baumannii* penicillin-binding protein 3 (PBP3). A molecular model of the acyl-enzyme complex of PBP3 (Protein Data Bank: 3UE3) (gray) with sulbactam (green) bound showing residues that were found to acquire substitutions during frequency of resistance determinations (cyan) as well as those amino acid substitutions identified in PBP3 from clinical isolates of *Acinetobacter* species (yellow) that had elevated sulbactam-durlobactam minimum inhibitory concentrations. The amino acids Ser390 and Thr526 are within hydrogen bonding distance of sulbactam.

Sulbactam-durlobactam was evaluated for its ability to lead to the in vitro emergence of resistance in clinical isolates of *Acinetobacter* species [29]. The frequency of resistance was determined to be 7.6 × 10^−10^ to <9.0 × 10^−10^ at 4 × MIC [29]. Stable mutants were whole genome sequenced to identify the mechanisms leading to sulbactam-durlobactam resistance. Most of the mutations mapped to *ftsI*, the gene that encodes PBP3, the target for sulbactam [29]. The corresponding amino acid substitutions identified, S390T, V505L, and T511A were near the active site serine (Ser336) (Figure 2), and the S390T variant was found to be approximately 140-fold less inhibited by sulbactam compared to wild-type PBP3 [29]. Mutations in tRNA synthetases, *aspS* and *gltX* were also identified in sulbactam-durlobactam–nonsusceptible *Acinetobacter* isolates. Mutations in these genes have been linked to induction of the stringent response, which is a cellular stress response that renders PBP2 dispensable [29]. In a separate study of 72 well-characterized *A. baumannii* isolates from the Walter Reed Army Medical Center, 4 isolates were found to have elevated sulbactam-durlobactam MICs (≥8 µg/mL) [24]. These isolates with elevated sulbactam-durlobactam MIC values encode either the A326V or S1010R substitutions in AdeJ, an efflux pump membrane transporter subunit, or for the H370Y or A578T amino acid substitutions in PBP3; however, these PBP3 residues sit further away from the active site and their impact on the ability of sulbactam to inhibit PBP3 has yet to be defined (Figure 2) [24]. In addition, results from another study suggested that durlobactam could be a substrate for select efflux pumps within *Acinetobacter* species as efflux pump knockout strains (Δ*adeB*, Δ*adeJ*, and Δ*adeB*/Δ*adeJ*) were more susceptible to sulbactam-durlobactam, but not sulbactam alone [24]. Overall, several studies have shown that in vitro spontaneous resistance to sulbactam-durlobactam is low. Notably, some strains that were resistant had a compensatory loss in fitness in vitro [29].

## IN VITRO ACTIVITY OF SULBACTAM-DURLOBACTAM AGAINST CONTEMPORARY ISOLATES OF THE *ACINETOBACTER BAUMANNII–CALCOACETICUS* COMPLEX

Several reports describe the in vitro antibacterial activity of sulbactam-durlobactam and comparator antibacterial agents against contemporary clinical isolates of the *Acinetobacter baumannii–calcoaceticus* complex (ABC) [30–35]. ABC is a closely related group of bacterial species that are often hard to differentiate from one another and are comprised of several species including *A. baumannii*, *A. calcoaceticus*, *A. nosocomialis*, and *A. pittii* [36]. The largest study to date evaluated 5032 ABC isolates collected between 2016 and 2021 from community- and hospital-associated infections in 33 countries across Asia/South Pacific (13.6%), Europe (42.1%), Latin America (12.6%), the Middle East (Israel only) (1.7%), and North America (United States only) (29.9%) [35]. Since *A. baumannii* is the ABC species most often associated with nosocomial outbreaks and high levels of antibacterial resistance, 80.2% of the isolates selected for this study were *A. baumannii*, followed by 12.7% *A. pittii*, 5.9% *A. nosocomialis*, and 1.1% *A. calcoaceticus*. Isolates were collected from 5 common infection sources: respiratory tract (54.3%), bloodstream (20.2%), urinary tract (16.5%), skin and soft tissue (4.5%), and intra-abdominal (4.3%). These distributions of isolates across species, geographic regions, and infection types were consistent for all 6 years of the study [35]. Against all 5032 ABC isolates, the addition of durlobactam to sulbactam lowered the MIC_90_ by 32-fold, compared to that of sulbactam alone, from 64 µg/mL to 2 µg/mL (Table 2).

**Table 2.** In Vitro Activity of Sulbactam-durlobactam and Comparator Antibacterial Agents Against Global *Acinetobacter baumannii–calcoaceticus* Complex Clinical Isolates Collected From 2016 to 2021

Category	Antibacterial Agent	No.	MIC_50_, µg/mL	MIC_90_, µg/mL	CLSI Interpretive Criteria^a^		
					% Susceptible	% Intermediate	% Resistant
All isolates	Sulbactam-durlobactam	5032	1	2	98.3	NA	1.7
	Sulbactam	…	8	64	46.9	8.0	45.1
	Amikacin	…	4	>64	58.6	3.3	38.1
	Cefepime	…	16	>16	44.6	7.9	47.4
	Ciprofloxacin	…	>4	>4	44.4	0.7	54.9
	Colistin	…	0.5	1	NA	95.9	4.1
	Imipenem	…	8	>64	48.9	0.6	50.5
	Meropenem	…	16	>64	47.9	1.1	51.0
	Minocycline	…	0.5	16	78.3	10.1	11.6
	Tigecycline	…	0.5	2	NA	NA	NA
*Acinetobacter* species							
*ȃA. baumannii*	Sulbactam-durlobactam	4038	1	2	98.0	NA	2.0
	Imipenem	…	32	>64	37.7	0.6	61.6
*ȃA. calcoaceticus*	Sulbactam-durlobactam	55	0.5	1	100	NA	0.0
	Imipenem	…	0.25	0.25	100	0.0	0.0
*ȃA. nosocomialis*	Sulbactam-durlobactam	296	0.5	1	99.7	NA	0.3
	Imipenem	…	0.25	0.5	92.2	0.0	7.8
*ȃA. pittii*	Sulbactam-durlobactam	636	0.5	2	99.4	NA	0.6
	Imipenem	…	0.25	0.5	95.1	0.3	4.5
Region							
*ȃ*Asia/South Pacific	Sulbactam-durlobactam	685	1	2	98.4	NA	1.6
	Imipenem	…	32	>64	43.9	0.4	55.6
*ȃ*Europe	Sulbactam-durlobactam	2121	1	4	98.6	NA	1.4
	Imipenem	…	32	>64	43.9	0.5	55.5
*ȃ*Latin America	Sulbactam-durlobactam	632	1	2	95.3	NA	4.7
	Imipenem	…	64	>64	28.8	0.2	71.0
*ȃ*Middle East (Israel)	Sulbactam-durlobactam	88	1	2	97.7	NA	2.3
	Imipenem	…	32	64	28.4	0	71.6
*ȃ*North America (US)	Sulbactam-durlobactam	1506	1	2	99.2	NA	0.8
	Imipenem	…	0.25	64	67.9	0.9	31.3
Infection type							
*ȃ*Respiratory tract	Sulbactam-durlobactam	2731	1	2	98.1	NA	1.9
	Imipenem	…	32	>64	43.3	0.6	56.1
*ȃ*Bloodstream	Sulbactam-durlobactam	1015	1	2	98.4	NA	1.6
	Imipenem	…	2	>64	50.9	0.6	48.5
*ȃ*Urinary tract	Sulbactam-durlobactam	832	1	2	98.9	NA	1.1
	Imipenem	…	0.5	64	62.7	0.6	39.7
*ȃ*Intra-abdominal	Sulbactam-durlobactam	217	1	2	97.7	NA	2.3
	Imipenem	…	32	>64	41.9	0.0	58.1
*ȃ*Skin and soft tissue	Sulbactam-durlobactam	227	1	2	99.1	NA	0.9
	Imipenem	…	0.5	>64	63.0	0.4	36.6

Source: [35].

Abbreviations: CLSI, Clinical and Laboratory Standards Institute; MIC_50_, The antibiotic concentration that inhibits the growth of 50% of the tested isolates; MIC_90_, The antibiotic concentration that inhibits the growth of 90% of the tested isolates; NA, not available; US, United States.

As published by CLSI M100 (2021) [39]. Sulbactam-durlobactam MICs were interpreted using the preliminary breakpoint of susceptible ≤4 µg/mL and resistant ≥8 µg/mL. Sulbactam MICs were interpreted using the sulbactam component of the ampicillin-sulbactam breakpoints (≤8/4 µg/mL [susceptible], 16/8 [intermediate], ≥32/16 [resistant]) [39].

**Figure 3.**
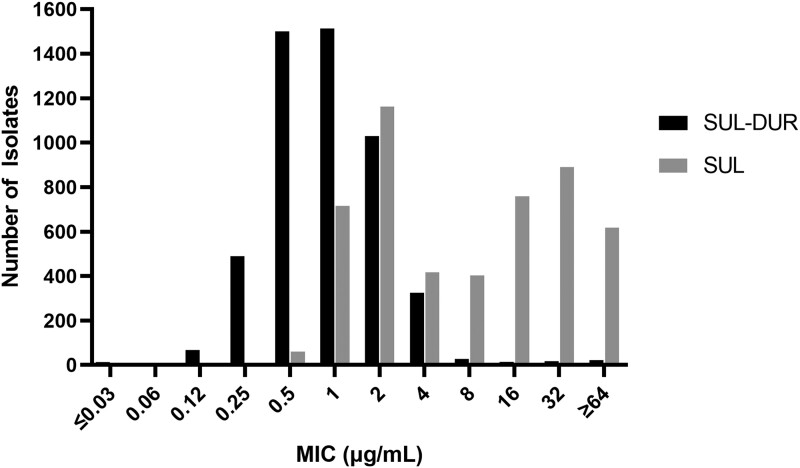
Minimum inhibitory concentration (MIC) distribution for sulbactam-durlobactam (SUL-DUR) and sulbactam (SUL) against 5032 *Acinetobacter baumannii–calcoaceticus* complex global isolates collected from 2016 to 2021 [35].

Using the preliminary sulbactam-durlobactam breakpoint of 4 µg/mL [37], 98.3% of the isolates were susceptible to sulbactam-durlobactam (Figure 3). Conversely, more than half of these isolates were nonsusceptible to carbapenems (51.1% and 52.1% for imipenem and meropenem, respectively). The colistin and tigecycline MIC_90_ values had similar potency to sulbactam-durlobactam; however, the in vitro susceptibilities of these agents often do not correlate with efficacy due to toxicities and poor pharmacokinetic properties [38].

The activity of sulbactam-durlobactam was consistent across the ABC species tested, despite large variations in carbapenem susceptibility (∼38% for *A. baumannii* isolates and ≥92% for the other species tested) (Table 2) [35]. The activity of sulbactam-durlobactam was also consistent across the geographical regions, with isolates from Europe having a MIC_90_ value 1 doubling dilution higher compared to the entire set (4 µg/mL vs 2 µg/mL). Across each region, the percentage of isolates with a sulbactam-durlobactam MIC ≤4 µg/mL was >95%. In addition, the potency of sulbactam-durlobactam was similar for each year (2016–2021) and different infection sources. Sulbactam-durlobactam was found to be active against phenotypically different resistant subsets, including carbapenem-nonsusceptible, colistin-resistant, multidrug-resistant (MDR), and extensively drug-resistant (XDR) isolates, each with an MIC_50_ of 1 or 2 µg/mL and an MIC_90_ of 4 µg/mL (Table 3). The percentage of isolates with a sulbactam-durlobactam MIC ≤4 µg/mL was >96% across these different antibiotic-resistant groups [35].

**Table 3.** In Vitro Activity of Sulbactam-durlobactam Against Phenotypically Antibiotic-Nonsusceptible *Acinetobacter baumannii–calcoaceticus* Complex Clinical Isolates Collected Globally From 2016 to 2021

Resistance Phenotype^a^	No.	Sulbactam-durlobactam		
		MIC_50_, µg/mL	MIC_90_, µg/mL	% Susceptible^b^
All isolates	5032	1	2	98.3
Sulbactam nonsusceptible	2670	1	4	96.9
Imipenem nonsusceptible	2570	1	4	96.7
Colistin resistant	204	2	4	98.0
Ciprofloxacin nonsusceptible	2796	1	4	97.5
Amikacin nonsusceptible	2083	2	4	96.9
Minocycline nonsusceptible	1092	2	4	97.7
Multidrug-resistant^c^	2680	1	4	96.9
Extensively drug-resistant^c^	2116	2	4	97.2

Source: [35].

Abbreviations: MIC_50_, The antibiotic concentration that inhibits the growth of 50% of the tested isolates; MIC_90_, The antibiotic concentration that inhibits the growth of 50% of the tested isolates.

Nonsusceptibility or resistance as determined by Clinical and Laboratory Standards Institute M100 (2021) [39].

Sulbactam and sulbactam-durlobactam MICs were interpreted using the preliminary breakpoint of susceptible ≤4 µg/mL.

Multidrug-resistant isolates were defined as nonsusceptible to 1 agent from ≥3 different antimicrobial classes and extensively drug-resistant isolates were defined as not susceptible to at least 5 of the following 7 agents (classes): cefepime (extended-spectrum cephalosporins), imipenem (carbapenems), amikacin (aminoglycosides), ciprofloxacin (fluoroquinolones), minocycline (tetracyclines), sulbactam (penicillin/β-lactamase inhibitor; sulbactam is the active component of ampicillin-sulbactam against *Acinetobacter* spp), and colistin (polymyxins), based on proposed international guidelines [40]. For colistin, only colistin-resistant isolates were used in these determinations as only intermediate and resistant interpretive criteria exist for colistin [39].

In addition to the 6-year global surveillance study [35], the in vitro activity of sulbactam-durlobactam was evaluated in a surveillance of 982 *A. baumannii* clinical isolates collected during 2016–2018 from 22 sites distributed across mainland China [32]. Isolates were mostly from hospital-associated infection types (89.5%) and 72.8% were from lower respiratory tract, 17.3% from intra-abdominal, 6.0% from urinary tract, 3.6% from skin and soft tissue, and 0.3% from bloodstream infections. For these isolates, susceptibility was low for levofloxacin (14%), imipenem (15.2%), and amikacin (26.2%). The MIC_50_ and MIC_90_ values of 1 and 2 µg/ml, respectively, for sulbactam-durlobactam, were 32-fold lower than those observed for sulbactam alone. This level of activity was consistent across Chinese sites and most infection types; however, the urinary tract isolates were more susceptible to sulbactam-durlobactam (MIC_50_ and MIC_90_ values of 0.5 and 1 µg/mL, respectively).

In 4 separate studies, sulbactam-durlobactam was profiled against collections of molecularly characterized, carbapenem-resistant *A. baumannii* clinical isolates from different geographical regions, which further demonstrates that durlobactam can restore sulbactam activity against MDR isolates from around the globe. These isolates represent a wide breadth of international clonal groups, presence of class D carbapenemases (eg, OXA-23–like, OXA-24/40–like, OXA-143–like, and OXA-58), and antibiotic resistance phenotypes. Nodari and colleagues tested 112 *A. baumannii* clinical isolates from Brazil that represent the major South American clones (mostly IC5, IC1, IC4, and IC7) for susceptibility to sulbactam-durlobactam [33]. All isolates were carbapenem-resistant and 92.8%, 86.6%, and 18.8% were amikacin-resistant, gentamicin-resistant, and polymyxin-resistant, respectively. Sulbactam-durlobactam MIC values ranged from ≤0.25 µg/mL to 4 µg/mL with MIC_50_ and MIC_90_ values of 1 µg/mL each, which was consistent across the different international clones and isolates expressing different class D carbapenemases. In Petropoulou et al, 190 unique carbapenem-resistant *A. baumannii* isolates collected from 11 hospitals in Greece during 2015 were assayed for susceptibility to sulbactam-durlobactam [30]. This collection displayed high levels of resistance to antibiotics with MIC_90_ values of 16 µg/mL for colistin, 32 µg/mL for minocycline, >64 µg/mL for imipenem, and >128 µg/mL for amikacin. Sulbactam-durlobactam had an 8-fold lower MIC_50_/MIC_90_ value of 4/8 µg/mL compared to sulbactam alone, with 87% of isolates with a sulbactam-durlobactam MIC value of ≤4 µg/mL.

One collection curated by researchers at the University of Fribourg, Switzerland, was comprised of 100 clinical *A. baumannii* isolates of worldwide origin and included producers of OXA-23 OXA-40–like, OXA-58, OXA-72, and NDM as well as 9 colistin-resistant isolates [34]. This set had a high degree of antibiotic resistance with susceptibility of only 18% for amikacin, 5% for cefepime, 0% for imipenem, 46% for minocycline, and 8% for sulbactam-cefoperazone. In contrast, 71% of isolates had a sulbactam-durlobactam MIC ≤4 µg/mL, the preliminary breakpoint [37]. Sulbactam-durlobactam showed little activity against the NDM-producing isolates, consistent with the finding that durlobactam does not inhibit metallo-β-lactamases [23].

The activity of sulbactam-durlobactam was also measured against 246 carbapenem-resistant *A. baumannii* isolates collected by researchers at the University of Cologne, Germany, between 2012 and 2016 from patients from 37 countries in 6 world regions (Africa, Asia/South Pacific, Europe, Latin America, Middle East, and North America) [31]. In addition to being resistant to carbapenems, 69.5% of isolates were resistant to amikacin, 24.4% resistant to minocycline, and 4.1% resistant to colistin. Sulbactam alone had MIC_50_/MIC_90_ values of 16/64 µg/mL. Conversely, sulbactam-durlobactam had MIC_50_ and MIC_90_ values of 1 and 2 µg/mL, respectively. There was no correlation between the sulbactam-durlobactam MICs and class D β-lactamases, other serine carbapenemases, or clonal strain type.

## EFFICACY OF SULBACTAM-DURLOBACTAM IN PRECLINICAL MODELS OF *A. BAUMANNII* INFECTION

Sulbactam-durlobactam has shown robust in vivo efficacy in multiple preclinical models of *A. baumannii* infection [23, 24]. The MDR and XDR isolates tested in these models had established drug resistance phenotypes (specifically class D β-lactamase production), with sulbactam-durlobactam MIC values ranging from 0.5 to 4 µg/mL. Treatment with sulbactam-durlobactam resulted in a dose-dependent reduction in XDR *A. baumannii* bacterial counts in both neutropenic mouse thigh abscess and pneumonia infection models, resulting in >1-log reduction of bacterial burden compared to the initial inoculum and multiple logs as compared to the growth control [41].

## CONCLUSIONS

Taken together, these results demonstrate that the biochemical inhibition of β-lactamases by durlobactam, as well as the restoration of in vitro susceptibility of sulbactam by durlobactam that was observed in multiple surveillance studies, is consistent with the robust in vivo efficacy of the combination seen in standard preclinical murine models of infection [41]. If approved, sulbactam-durlobactam may address an urgent unmet medical need for patients with serious infections caused by *Acinetobacter* species, including MDR strains.

## Notes

***Acknowledgments.*** The effort for this review was supported in part by funds and/or facilities provided by the Cleveland Department of Veterans Affairs (VA Merit Review Award Number 1I01 BX002872 to K. M. P.-W. from the Biomedical Laboratory Research and Development Service of the VA Office of Research and Development).

***Financial support.*** This work was sponsored by Entasis Therapeutics, Inc., a wholly owned subsidiary of Innoviva, Inc. The authors did not receive any fees for authorship.

***Supplement sponsorship.*** This article appears as part of the supplement “Sulbactam-durlobactam, a Targeted β-lactam/β-lactamase Inhibitor, for MDR *Acinetobacter*,” sponsored by Entasis Therapeutics Inc., a wholly owned subsidiary of Innoviva, Inc.
